# Detection of Development-Specific MicroRNAs in Rabbit Embryos and Culture Media: A Potential Biomarker Approach for Embryo Quality Assessment

**DOI:** 10.3390/genes16091042

**Published:** 2025-09-03

**Authors:** María Salinas, Nikolett Tokodyné Szabadi, Gréta Dévai, Martin Urbán, Arnold Tóth, Bence Lázár, Timea Pintér, Annamária Nemes, Péter Fancsovits, Lilla Bodrogi, Elen Gócza

**Affiliations:** 1 Institute of Genetics and Biotechnology, Animal Bio-Technology Department, Hungarian University of Agriculture and Life Sciences, 2100 Gödöllő, Hungary; mtsalinasaponte@gmail.com (M.S.); tokodyne.szabadi.nikolett@uni-mate.hu (N.T.S.); greta.devai@gmail.com (G.D.); urban.martin@uni-mate.hu (M.U.); toth.arnold94@gmail.com (A.T.); lazar.bence@uni-mate.hu (B.L.); timea.pinter90@gmail.com (T.P.); bodrogi.lilla@uni-mate.hu (L.B.); 2 Agribiotechnology and Precision Breeding for Food Security National Laboratory, 2100 Gödöllő, Hungary; 3 National Centre for Biodiversity and Gene Conservation, Institute for Farm Animal Gene Conservation, 2100 Gödöllő, Hungary; 4 Division of Assisted Reproduction, Department of Obstetrics and Gynaecology, Semmelweis University, 1085 Budapest, Hungary; nemes.annamaria@semmelweis.hu (A.N.); fancsovits.peter@semmelweis.hu (P.F.)

**Keywords:** microRNA, blastocyst, IVF, embryo viability, biomarker

## Abstract

MicroRNAs (miRNAs) are short, non-coding RNA molecules that play a crucial role in regulating various biological processes by influencing post-transcriptional gene expression and gene silencing. **Background/Objectives**: In this study, rabbit embryos were utilised as a model system to investigate potential biomarkers relevant to human embryo development. Seven microRNAs (miRNAs) identified in the embryo culture medium were evaluated as biomarkers by analysing the correlation between their expression levels and the developmental quality of rabbit embryos at days 4 and 6. **Methods**: We analysed the expression of seven development-specific miRNAs (miR-24-3p, miR-28-3p, miR-103a-3p, miR-181a-5p, miR-191-5p, miR-320a-3p, miR-378a-3p) in 4-day-old and 6-day-old rabbit embryos, along with their culture media. **Results**: Our findings revealed significant differences in the expression levels of these miRNAs between the 4-day-old and 6-day-old embryos. On the other hand, the expression patterns observed in the culture medium samples showed less variation between the two age groups. Nonetheless, analysis of miRNA expression profiles in the spent culture medium from individually cultured embryos enabled the identification of lower-quality embryos, characterised by smaller size and impaired or delayed development. **Conclusions**: The detection of these miRNAs in embryo culture medium may serve as a reliable indicator of successful progression to the blastocyst stage. Our experimental results identified specific miRNAs whose expression profiles differ according to embryonic stage and quality, thereby reflecting key developmental milestones. Notably, the detectability of these miRNAs in the medium—without prior RNA isolation—indicates their active secretion into the extracellular environment. By synthesising our findings with the existing literature, we refined a panel of miRNAs essential for the development of implantation-competent embryos in both rabbits and humans. Consequently, we developed a non-invasive assay for predicting implantation and pregnancy outcomes, which may have significant applications in human reproductive medicine.

## 1. Introduction

In recent years, miRNAs have become popular in experimental research. These small molecules have shown huge importance in several biological processes, specifically embryonic development, where stem cells undergo many differentiation and proliferation steps [1].

miRNA are small non-coding RNA molecules, which principally repress the expression of genes by binding to the 3′UTR of target mRNAs, leading to inhibition of mRNA translation or mRNA [1,2].

Several studies where biogenesis of miRNA has been blocked show the importance of miRNAs supporting embryo development and embryonic stem cell features, as well as studies of miRNA inhibition, miRNA overexpression, transcriptomic analysis and sequencing have shown the function of specific miRNA [2,3]

According to Omes (2024), hsa-miR-661 is considered as a negative regulator which participates in organelle assembly and cell shape and cytoskeletal structure [4], hsa-miR-21-5p is involved in cell proliferation, driving embryo competence and embryo viability [4,5].

Bta-miR-34c participates actively in the fertilisation of oocytes and development into a blastocyst. It has been related to the high quality of the embryos in bovine, improving the high total cell number and inner cell mass and the time of embryonic genome activation [6]. Bta-miR-141 has been found conserved in vertebrates. In bovines, it is known for its role in oocyte maturation and oocyte competence. In addition, bta-miR-141 is also participating in the regulation of cell differentiation and cell division in early embryonic development [6].

Bta-miR-378a-3p has been found in embryonic extracellular vesicles (EVs), excreted from blastocysts; it is known that this miRNA has an important role in blastocyst hatching in bovine by modulating relevant signalling pathways and playing a role in intercellular communication within embryos and maintaining miRNA homeostasis with surrounding cells. In addition, transcriptomic research with overexpression and inhibition of bta-miR-378a-3p confirmed the relationship with numerous genes related to signalling, metabolism and development [7].

Research carried out by Turri, F (2021) in bulls showed that high expression of miR-378 is found in high-quality sperm and improved fertility-related sperm. High expression of miR-2285n is associated negatively with sperm motility and fertility [8]. In the same way, in human, hsa-miR-378a-3p has been related to the regulation in oocyte meiosis, which produces viable oocytes and high expression of hsa-miR-378a-3p was linked to successful implantation [9].

Kamijo, S (2022) compared the expression of miRNAs in media from successful implantation human embryos and non-successful implantation embryos. Hsa-miR-16-5p, hsa-miR-30e-5p, hsa-miR-320a, hsa-miR-320b, hsa-miR-509-3p, hsa-miR-99b-5p and hsa-let-7c-5p were found highly expressed in the media of non-successful implantation embryos media while hsa-miR-148a-3p, hsa-miR-181a-5p, hsa-miR-192-5p, hsa-miR-28-3p, hsa-miR-371a-5p, hsa-miR-378a-3p, hsa-miR-373-3p and hsa-let-7i-5p were associated with better embryonic outcomes and are believed to play roles in embryonic maturation and implantation processes [9].

Finding miRNA in a medium which was used to culture embryos suggests that the measurement of the expression of certain miRNAs can predict embryo development and implantation potential in assisted reproduction methods, making these miRNAs potential biomarkers to support in vitro fertilisation IVF [4,7,9].

We analysed the expression of seven embryo-specific miRNAs (miR-24-3p, miR-28-3p, miR-103a-3p, miR-181a-5p, miR-191-5p, miR-320a-3p, and miR-378a-3p) in 4-day-old and 6-day-old rabbit embryos, as well as in their culture media. By comparing the miRNA expression profiles of these miRNAs in the culture medium collected from individually cultured embryos, we could identify differences in developmental stages and lower-quality embryos.

## 2. Materials and Methods

**Figure 1:**
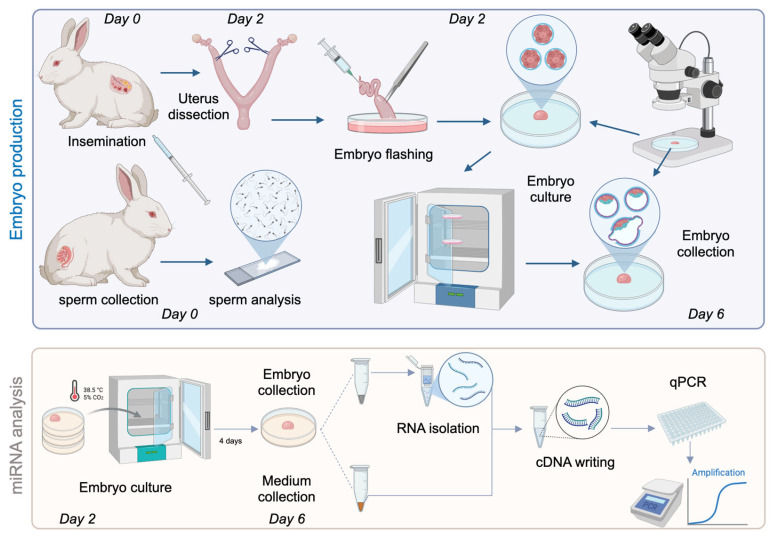
Schematic flowchart summarising the steps of the experimental process.

The methodology comprised four main steps: embryo production, embryo culture, miRNA amplification, and miRNA analysis, as illustrated in Figure 1.

### 2.1. miRNA Selection

The examined miRNAs were chosen based on a literature review. We determined the rabbit-specific miRNA sequences based on our earlier research [10], (Table 1).

### 2.2. Experimental Animals and Animal Care

The New Zealand white rabbits (*Oryctolagus cuniculus*) were provided by Innovo Ltd, Isaszeg, Hungary. All procedures involving animals were conducted in accordance with the relevant European legislation on the protection of animals used for scientific purposes, specifically Directive 2010/63/EU of the European Parliament and of the Council. It was approved by the Pest County Government Office’s Directorate of Food Chain Safety, under the permit number PE/EA/00741-7/2022.

The animals were housed at the Institute of Genetics and Biotechnology, Hungarian University of Agriculture and Life Sciences (MATE), in a climate-controlled animal facility maintained at a constant temperature of 19 °C. Continuous veterinary supervision was ensured throughout the study. Ad libitum access to feed (Purina Mills, LLC (Purina Animal Nutrition), Gray Summit, MO, USA) and drinking water, and environmental enrichment in the form of chew sticks was provided. A 24 h artificial light–dark cycle was maintained, consisting of 16 h of light and 8 h of darkness daily.

### 2.3. Rabbit Embryo Collection and Cultivation

Female rabbits aged 16–20 weeks (mean body weight: 3.0–3.5 kg) were artificially inseminated using freshly collected sperm from male rabbits, aged ≥ 18–20 weeks. Prior to use, sperm samples were examined microscopically to confirm sufficient motility. Ejaculate volume was measured using a precision pipette. Sperm motility was subjectively assessed under light microscopy (Leica Microsystems GmbH, Wetzlar, Germany). Sperm concentration was determined using a Makler counting chamber after a 1:4 dilution in distilled water, which immobilised sperm through hypotonic lysis. Concentrations were calculated from counts in 30 grid squares. Semen from four males was collected during each experiment, and only samples of acceptable quality were pooled prior to insemination. One-cell stage embryos were collected 20 h post-insemination, while eight-cell stage embryos were retrieved 38–40 h post-insemination.

Prior to embryo collection, all donor females were euthanized with 0.25 mL/kg BW Euthanimal (Alfasan Nederland B.V., Utrecht, Netherlands) and dissected under sterile conditions. The ovaries, fallopian tubes, and cranial segments of the uterine horns from both sides were excised and transferred into Petri dishes containing Dulbecco’s phosphate-buffered saline pre-warmed to 38.5 °C.

For embryo recovery, the oviduct and adjacent uterine horn were flushed with a pre-heated medium using a 20 mL sterile syringe equipped with a 19G injection needle (Figure 1). The flushing medium consisted of 79% DPBS, 20% heat-inactivated foetal bovine serum, and 1% Penicillin-Streptomycin, and was filtered through a 0.22 μm membrane filter prior to use. The flushing was performed at 38.5 °C, and the effluent was collected in three individual 35 mm Petri dishes maintained at the same temperature.

The number of corpora hemorrhagica visible on the ovarian surface was counted to estimate the expected embryo yield from each donor.

Recovered embryos were washed through three successive drops of G-TL human embryo culture medium (Cat. No. 10145, Vitrolife, Göteborg, Sweden) supplemented with hyaluronan and human serum albumin. Embryos were subsequently transferred to fresh G-TL medium droplets, pre-equilibrated under sterile mineral oil (OVOIL, Cat. No. 100290, Vitrolife, Göteborg, Sweden) and cultured in an incubator set at 38.5 °C in a humidified atmosphere of 5% CO_2_.

In the second part of the experiment, eight-cell-stage embryos were individually cultured in separate drops. The embryo and medium samples were collected on Day 6. For time-lapse imaging, embryos were washed out on Day 2 and individually cultured in a separate drop of GT-L medium until reaching the expanded blastocyst stage. Time-lapse microscopy analysis was begun in the afternoon on Day 2. Embryonic development was monitored using the CytoSMART Lux2 system (Lonza Group Ltd., Basel, Switzerland). Images were acquired at five-minute intervals under standard culture conditions (Supplementary Movie S1).

### 2.4. Sample Collection, RNA Isolation, cDNA Writing and qPCR

The flushed embryos were morphologically analysed with a stereomicroscope (Leica M205FCA supplied with DFC7000-T Leica camera, Wetzlar, Germany) and cultured in vitro for 4 or 6 days in an incubator at 38.5 °C and 5% CO_2_.

A quantity of 9 μL of medium (G-TL human culture medium) per embryo was taken into clean microcentrifuge tubes. We transferred the embryos into tubes containing 100–100 µL of RNA Aqueous Lysis Buffer Micro Kit (Applied Biosystems™ (brand under Thermo Fisher Scientific, Foster, CA, United States), containing guanidinium thiocyanate.

The samples were stored at −80 °C until RNA isolation in the case of embryo samples and cDNA transcription in the case of medium samples.

RNA isolation was performed just for embryo samples with RNAqueous™-Micro Total RNA Isolation Kit AM1931 (Applied Biosystems™). The isolated RNA was checked using a NanoDrop (ND-1000, Thermo Fisher Scientific, Waltham, MA, USA, UV-Vis). While medium samples were taken directly to cDNA writing without RNA isolation, it was expected that miRNAs would already be in the medium.

Samples were written into cDNA using the Applied Biosystems TaqMan Advanced miRNA cDNA Synthesis Kit (catalogue number: A28007). The concentration of RNA and culture medium samples was checked (Nano Drop) during the experiment setup, as the sample concentration should be 5 ng/µL before addition to the reactions. Due to the low concentrations, the maximum sample volume (2 µL) was used throughout the experiment without dilution. The workflow of cDNA synthesis consists of four steps: the first step, the poly (A) tailing reaction, after the adapter ligation reaction, then the reverse transcription (RT) reaction, and perform the miR-Amp reaction. Each step was performed according to the protocol included with the kit, paying attention to important notes (for example: dissolving 50% PEG 8000 reagent) and volumes. Until real-time PCR was performed, the undiluted miR-Amp reaction product was stored at –80 °C until use.

TaqMan™ Fast Advanced Master mix (Applied Biosystems™) (catalogue number: 4444963) qPCR was prepared with the following proportions for each primer (Table 2):

15 μL TaqMan™ Fast Advanced Master mix containing the corresponding miRNA primer was added to each position of the qPCR plate, and then 5 μL of the cDNA samples (1:10) were added, the cDNA samples were diluted 1:10 by adding RNase-free water for optimal real-time PCR reaction. Additionally, 4 μL of RNase-free water was added as well as a negative control.

For each sample, the reaction was performed in 3 parallel reactions for the tested miRNAs. miR-92 was used as an internal control (as housekeeping gene).

### 2.5. Data Analysis—MultiD GenEx

The obtained data were analysed with the MultiD GenEx qPCR data analysis software (Available online: http://www.multid.se (accessed on 1 May 2019), version 7.0). For comparing multiple groups, one-way ANOVA analysis of variance was used. Norm Finder tool, integrated within the GenEx software (version 7.0), was used to identify the most stable reference genes for normalisation in qPCR. According to this analysis we used the miR-92a-3p as an internal control. F3_medium pool was used as a reference sample during the data analysis.

The Kohonen and SOM (self-organising map) function was used to create a heatmap and groups which illustrates the difference between expressions and the correlations between samples.

### 2.6. SOLiD Sequencing

The libraries were prepared using total RNA samples previously isolated from rabbit embryos and embryonic rabbit fibroblasts sequenced at the Institute of Plant Genomics, Human Biotechnology and Bioenergetics of the Bay Zoltán Applied Research Foundation, Szeged, using the SOLiD 3 System (Applied Biosystems). The data files obtained during the sequencing are available at https://www.ebi.ac.uk/ena/data/view/ERP002216 (accessed on 11 September 2013) using the SRA Webin. The data obtained from SOLiD sequencing were analysed using the SOLiD System Small RNA Analysis Pipeline Tool (RNA2MAP, version 0.5), and the rabbit sequences were annotated based on known human (hsa-), bovine (bta-), and mouse (mmu-miRNA sequences downloaded from miRBase (Available online: https://mirbase.org (released 17: March 2011, accessed on 11 September 2013)).

## 3. Results

### 3.1. Sample Collection

We first analysed the expression of miRNAs in both groups of cultured embryos and their corresponding culture media. The first group, designated Pool A_II_e, consisted of five embryos, and the culture medium for these embryos was labelled A_II_m. The second and third samples, referred to as F1_m and F3_m, were derived from the culture media of embryo drops, each containing five embryos cultivated together. The developmental stages and morphological classifications at the time of RNA isolation for each sample are detailed in the accompanying Supplementary Table S1A.

In total, fifteen embryos were individually cultured, and we collected the culture media separately. In case of six embryos, we could analyse both the embryos and six related culture media, which was used for subsequent miRNA analysis. We analysed further nine culture media collected from individually cultivated embryos. The developmental stages and morphological classifications of each sample are provided in the accompanying Supplementary Table S1B.

### 3.2. miRNAs Expression Profile

This study aimed to correlate the quality of the embryo with the presence of certain miRNAs in the medium. The collected embryos were morphologically characterised (Supplementary Tables S1A and S1B) according to the embryo images. We examined the correlation in image-based categories with the related miRNA’s expression profile.

We checked the most relevant miRNAs according to the literature overview, and in our rabbit solid miRNA-based sequency database, constructed in our previous experiment [10]. We determined rabbit miRNA sequences by comparing human miRNA sequences published in miRBase (Available online: https://mirbase.org (released 17: March 2011, accessed on 11 September 2013)) as a reference (Table 1). We examined the abundance of these sequences in 6 and 7-day-old rabbit embryos (Supplementary Figure S1), as well as in rabbit (REF) and mouse embryonic fibroblasts (MEF), rabbit embryonic stem cells (ESC), and rabbit genital ridge (gonad) of 14-day-old embryos. Table 3 shows the read counts of different samples. We chose advanced hsa-miRNA primers, which the supplier offered, explicitly targeting the same type of rabbit miRNAs. We cultivated the rabbit embryos in GT-L medium, covered with 20 μL drops of OVOIL (Supplementary Movie S1) because we planned to translate our experimental background and apply the primers to analyse miRNA expression in the culture medium of in vitro–cultured human embryos in the future.

We examined the expression of twelve miRNAs in isolated RNA samples collected from rabbit embryos and medium drops. Has-miR-92a-3p and has-miR-320a-3p exhibited robust expression levels across nearly all embryo and medium samples analysed, indicating their potential roles in embryonic development and signalling. In contrast, the expression of mmu-miR-302b-3p and hsa-miR-371a-5p was undetectable throughout the analyses.

For mmu-miR-302b-3, we obtained low read numbers in the sequence library of 6-day-old and 7-day-old in vivo rabbit embryos, and we were unable to find counts of hsa-miR-302b-3p-related sequences in our database.

For hsa-371a-5p, according to solid sequencing results, we obtained high read numbers in 7-day-old in vivo rabbit embryos; however, it was undetectable throughout the analyses of in vitro cultured embryos. miR-28-3p was present only in later embryonic stages of in vivo developing rabbit embryos, as indicated by our solid sequencing data.

We used hsa-miR-92-3p as a reference gene, as we found constant and high expression examined all samples. Finally, we analysed the expression profiles of seven miRNAs.

For subsequent analysis, the embryos were organised into distinct groups.

#### 3.2.1. Comparative Analysis of miRNA Expression Profiles in the Blastocysts Pool and Their Culture Medium

**Figure 2:**
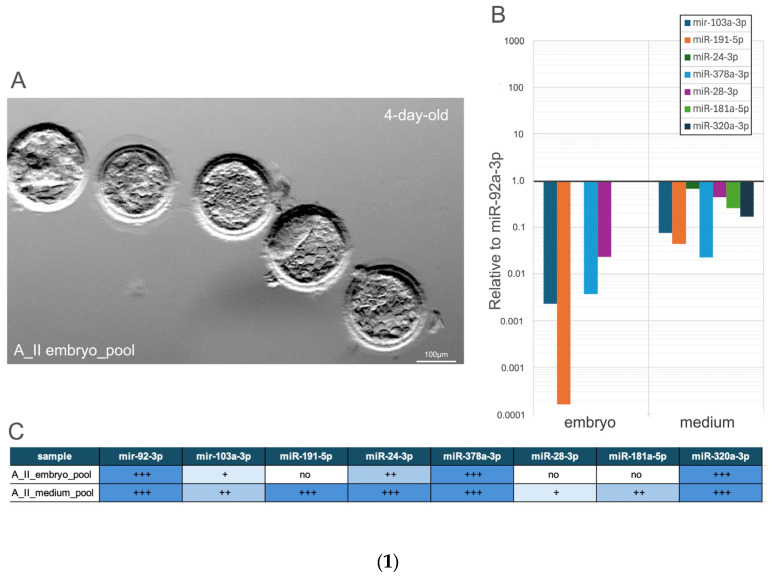

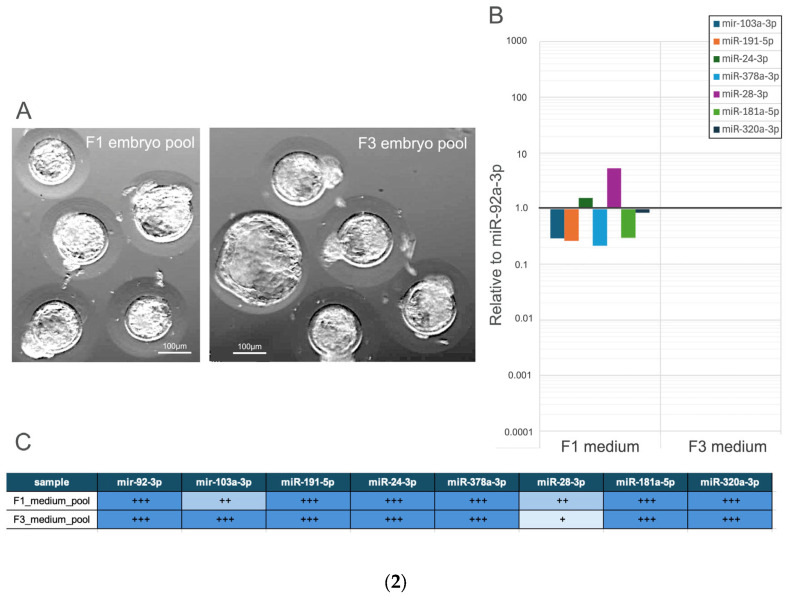
(**1A**): Representative images of the investigated embryos. (**1B**): Relative expression levels of the analysed microRNAs, normalised to miR-92a-3p as the reference gene, and used the F3_m culture medium sample as the reference sample, as all investigated miRNAs were detected in this pooled medium sample. Data are presented on a logarithmic scale to interpretation for variation in expression levels. (**1C**): Summary table of miRNA expression in different samples, categorised into four levels based on cycle threshold (Ct) values from quantitative real-time PCR. Ct values (+++: Ct < 35; ++: 35 < Ct < 39; +: 39 < Ct < 45; no expression: Ct > 45). (**2A**) Representative morphology of 6-day-old rabbit embryos from the F1 and F3 embryo pool. (**2B**) Relative expression levels of miRNA profile in embryo culture medium pools, normalised to miR-92a-3p. (**2C**) Summary of miRNA presence or absence in embryo and medium pools, based on qualitative expression.

Sample A_II comprised a pooled collection of five 4-day-old embryos (blastocyst stage 2) (Figure 2(1A)), along with the corresponding culture medium. The expression of seven miRNAs was analysed separately for both the embryos and the medium to detect differences in miRNA expression between the embryonic tissue and its surrounding environment.

All seven miRNAs were found in the medium samples (A_II_m, F1_m, F3_m).

The expression of hsa-miR-24-3p, hsa-miR-320a-3p, and hsa-miR-181-5p was detected only in the medium, indicating that these miRNAs are either secreted or less retained within the embryos. In contrast, the downregulated expression of hsa-miR-103a-3p, hsa-miR-378a-3p, hsa-miR-191-5p, and hsa-miR-28-3p in the medium was significantly higher compared to their expression levels in the embryos. MiR-92a-3p was used as an internal control, and the F3_medium pool served as a reference sample during data analysis, as all examined miRNAs were present in this sample.

#### 3.2.2. Comparative Analysis of miRNA Expression Profiles in Hatched Blastocysts and Their Culture Medium

**Figure 3:**
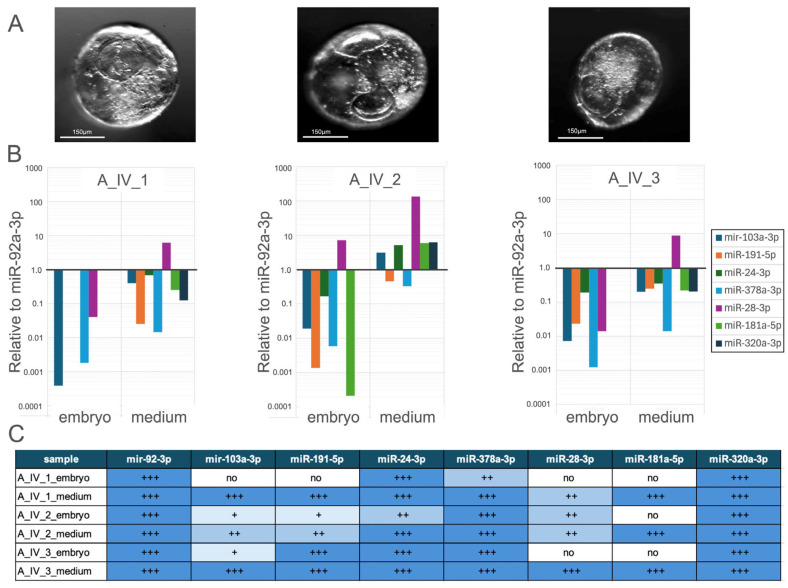
(**A**) Morphology of a rabbit hatched blastocyst. (**B**) Relative expression levels of miRNA profile in embryo and culture medium, normalised to miR-92a-3p. (**C**) Summary table of miRNA expression in different samples, categorised into four levels based on cycle threshold (Ct) values from quantitative real-time PCR. Ct values (+++: Ct < 35; ++: 35 < Ct < 39; +: 39 < Ct < 45; no expression: Ct > 45).

Three embryos categorised as hatched blastocysts (Figure 3A) were cultured individually and designated as A_IV_1, A_IV_2, A_IV_3. The expression levels of seven miRNAs were analysed independently in both the embryos and their corresponding culture media, as presented in Figure 3B,C.

In sample A_IV_1, seven miRNAs were detected as downregulated relative to miR-92, with the exception of hsa-miR-28-3p, whose expression was upregulated in the medium. hsa-miR-191-5p, hsa-miR-24-3p, hsa-miR-181-5p, and hsa-miR-320a-3p were not detected in A_IV_1 embryo. hsa-miR-103a-3p, hsa-miR-378a-3p, and hsa-miR-28-3p were detected in both samples; nevertheless, those miRNA expressions were higher in the medium than in the embryo sample.

In sample A_IV_2_embryo, all miRNAs were downregulated relative to the reference miRNA except for hsa-miR-28-3p, which was upregulated; additionally, hsa-miR-320a-3p was not detected. In A_IV_2_medium, the expression of all miRNAs was upregulated except for hsa-miR-191-5p and hsa-miR-378a-3p, whose expression levels were higher than those observed in A_IV_2_embryo. hsa-miR-24-3p high expression has been seen in both samples, and hsa-miR-181-5p was strongly downregulated in A_IV_2_embryo. hsa-miR-28-3p expression was found strongly upregulated in the medium.

In sample A_IV_3_embryo and A_IV_3_medium, miRNA expression remained downregulated, except hsa-miR-28-3p, which was upregulated in medium. hsa-miR-181-5p and hsa-miR-320a-3p were not detected in the embryo. hsa-miR-24-3p in both samples keeps balanced expression, while hsa-miR-378a-3p was strongly downregulated in embryo.

#### 3.2.3. Comparative Analysis of miRNA Expression Profiles in Individually Cultured Blastocysts and Their Culture Medium

**Figure 4:**
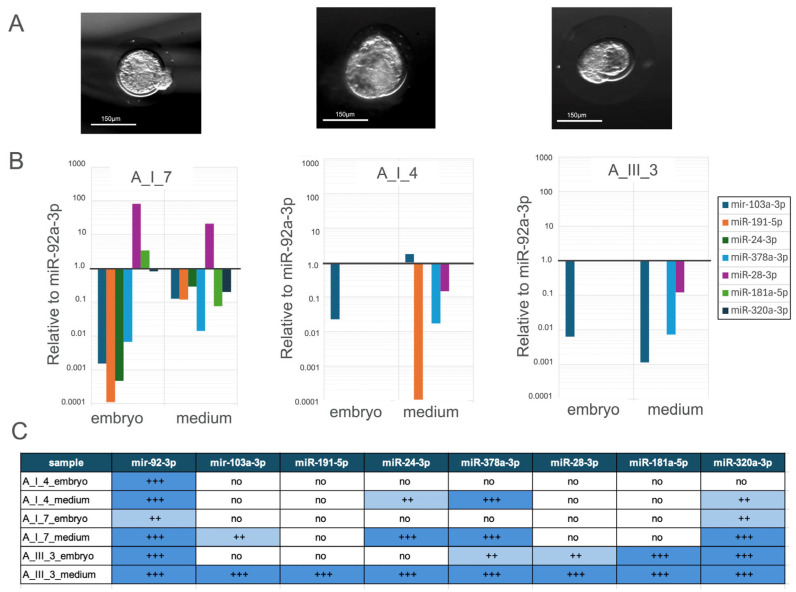
(**A**) Morphology of a rabbit blastocyst. (**B**) Relative expression levels of miRNA profile in embryo and culture medium, normalised to miR-92a-3p. (**C**) Summary table of miRNA expression in different samples, categorised into four levels based on cycle threshold (Ct) values from quantitative real-time PCR. Ct values (+++: Ct < 35; ++: 35 < Ct < 39; no expression: Ct > 45). In sample A_I_7_embryo, the expression of hsa-miR-103a-3p, hsa-miR-191-5p, has-miR-24-3p, hsa-miR-378a-3p, and hsa-miR-320a-3p was downregulated in the embryo and in medium; nevertheless, their expression in the medium is higher. The expression of hsa-miR-28-3p and hsa-miR-181-5p was upregulated in the embryo, and their expression was higher than that found in the medium.

Three embryos categorised as blastocysts (Figure 4A) were cultured individually and designated as A_III_3, A_I_4 and A_I_7. The expression levels of seven miRNAs were determined separately for both the embryos and their corresponding culture medium, as presented in Figure 4B,C.

In sample A_I_4_embryo just hsa-miR-103a-3p was detected and strongly found downregulated. In A_I_4_medium, the expression of hsa-miR-103a-3p was found upregulated, while just hsa-miR-191-5p, hsa-miR-378a-3p and hsa-miR-28-3p were detected slightly.

In sample A_III_3, hsa-miR-103a-3p was found downregulated in both samples. In the medium sample, only hsa-miR-181a-5p and hsa-miR-320a-3p were found downregulated.

#### 3.2.4. miRNAs Expression Profile—Kohonen’s Self-Organising Map (SOM)

Additionally, Kohonen’s Self-Organising Map (SOM) was applied to identify and visualise groups of samples exhibiting similar expression patterns.

**Figure 5:**
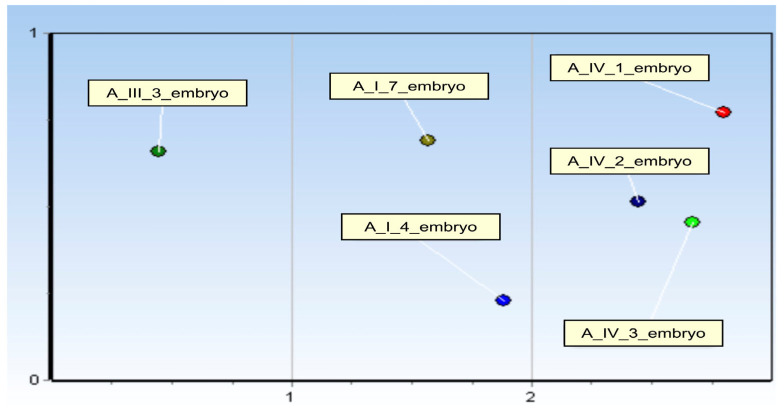
Kohonen Self-Organising Map Showing Clustering of Embryo Samples Based on miRNA Expression Profiles.

Additionally, the six samples (A_IV_1, A_IV_2, A_IV_3, A_I_4, A_I_7, and A_III_3) were grouped using Kohonen’s self-organising grouping SOM (Figure 5).

A_III_3 sample presented a unique miRNA profile, suggesting that it is developmentally distinct profile. A_I_7_embryo and A_I_4_embryo shared similar miRNA pattern. A_IV_1_embryo, A_IV_2_embryo and A_IV_3_embryo shared strong miRNA profile similarities.

#### 3.2.5. miRNAs Expression Profile—Heatmap

**Figure 6:**
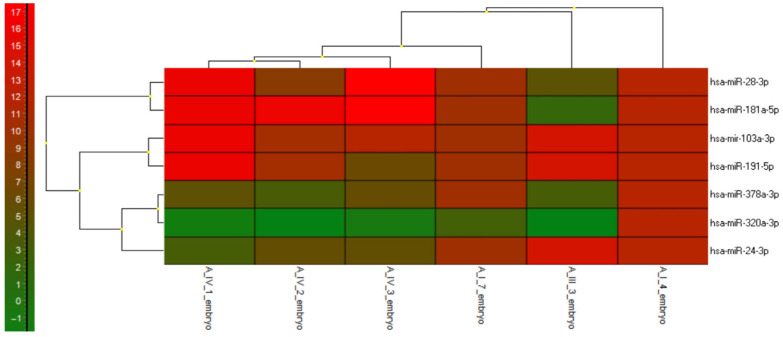
Heatmap and Clustering of miRNA Expression Across Rabbit Embryo Samples.

Six samples (A_IV_1, A_IV_2, A_IV_3, A_I_4, A_I_7, and A_III_3) were analysed using the ΔCt value to generate a heatmap visualisation (Figure 6).

The expression levels of hsa-miR-28-3p, hsa-miR-103a-3p, miR-181a-5p and hsa-miR-191-5p remained consistently high across most samples, suggesting that these miRNAs may have stable or essential functions in embryonic development. In contrast, hsa-miR-320a-3p and hsa-miR-378a-3p exhibited relatively low expression levels in most samples. hsa-miR-24-3p has shown moderate expression.

#### 3.2.6. Comparative Analysis of miRNA Expression Profiles in Samples and Their Culture Medium

**Figure 7:**
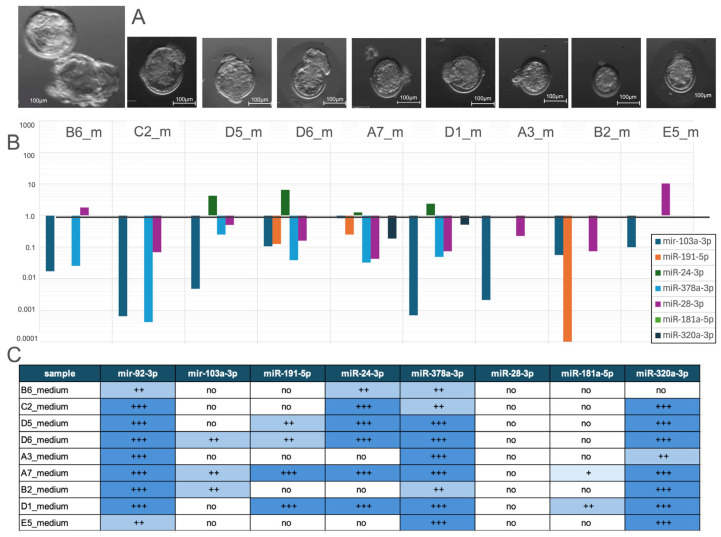
(**A**) Morphology of a rabbit hatched blastocyst. (**B**) Relative expression levels of miRNA profile in culture medium, normalised to miR-92a-3p. (**C**) Summary table of miRNA expression in different samples, categorised into four levels based on cycle threshold (Ct) values from quantitative real-time PCR. Ct values (+++: Ct < 35; ++: 35 < Ct < 39; +: 39 < Ct < 45; no expression: Ct > 45). The expression of hsa-miR-103a-3p, hsa-miR-28-3p, hsa-191-5p and hsa-miR-378a-3p was predominantly downregulated.

A total of 9 samples corresponding to hatched blastocysts stage 1 and stage 2 (Figure 7A) cultured media were analysed to assess their miRNA expression profiles (Figure 7B,C).

In contrast, hsa-miR-24-3p was found upregulated in 4 media. Notably, hsa-miR-181a-5p was undetected in most samples, and hsa-miR-320a-3p was detected in two samples.

## 4. Discussion

MicroRNAs have been implicated in key reproductive processes, including fertilisation, gametogenesis, embryo development, and implantation. Given the well-established roles of various miRNAs in embryonic development, research has increasingly focused on exploring their potential applications and practical uses in this research area [11]. Our results are consistent with other researchers demonstrating that several microRNAs are detectable in the culture medium of embryos, offering a promising approach for assessing embryo viability without invasive techniques [12,13,14].

miR-103a-3p moderate-high expression has been observed in the culture medium during our comparative analysis compared to its moderate expression in embryo samples. miR-103a-3p and members of in this group of miRNAs have been associated with cell proliferation [15], contributing to the understanding of embryonic stem cell regulation and function [16]. Furthermore, miR-103a-3p shows stability in porcine liver and uterus, making it suitable for normalisation in expression analysis [5]

Low-to-moderate miR-191-5p expression has been detected in the embryo and high expression in most of the medium samples. Several sources confirmed the expression of miR-191-5p in failed IVF, in aneuploid embryos and patients with cancer, suggesting its involvement in tumour [5,9,17]. miR-191-5p is correlated with successful pregnancy states, suggesting its role in supporting the developmental processes necessary for implantation and early embryogenesis, making it a good embryo viability and predicting implantation marker [18].

Mutia, (2023) describes the miR-24-3p role as a regulator of differentiation of ESCs by pluripotent markers Oct4, Nanog, Klf4, and c-Myc [11]. miR-24-3p was highly detected in most of the medium samples and in half of the embryo samples. Additionally, miR-24-3p contributes to the cell survival of porcine granulosa and overall follicular health, with regulatory effects on proliferation and apoptosis through its interaction with the target gene P27 [19].

miR-378a-3p was strongly present in most medium and embryo samples. bta-miR-378a-3p has been investigated and is known to enhance blastocyst quality, promote cell survival, and regulate embryo hatching. Similarly, hsa-miR-378a-3p has been associated with the regulation of oocyte meiosis, which is a vital process for the development of viable eggs and successful fertilisation. Pavani, (2022) linked the higher levels of hsa-miR-378a-3p to successful implantation outcomes [7,9].

miR-28-3p showed moderate expression levels in medium samples and in few embryo samples, suggesting a significant role in intracellular and extracellular regulation. Higher levels of hsa-miR-28-3p may be linked to successful implantation results and embryo development by various signalling pathways. In rabbits, the expression of miR-28-3p is high in good-quality blastocysts. Additionally, miR-28 may influence gene expression linked to cell proliferation, apoptosis, and metastasis [9,19].

High-moderate miR-181a-5p expression has been found in medium, suggesting that there is an active participation of miR-181a-5p in embryo regulation. During embryonic development, hsa-miR-181a-5p is involved in the oestrogen signalling pathway that influences embryo viability and implantation success. It is considered a key regulator of factors that affect embryo viability. miR-181a-5p is known to act suppressing or promoting (EMT) epithelial–mesenchymal transition by regulating molecules within pathways such as TGF-β, Wnt/β-catenin, and NF-κB, thereby influencing cancer cell invasion, migration, and stemness properties [9,20].

We used miR-92a-3p as an inner control gene. According to Caporali (2011) the overexpression of miR-92a induced severe defects in intersegmental vessel formation in zebrafish [21].

We found high miR-320a-3p expression in rabbit blastocysts and the culture media. It was published that miR-320 was in the top 5% most highly expressed miRNAs in the initial 9 euploid embryos; moreover, miR-320 target has been identified ITGB5, which plays a role in cell–matrix interaction [21,22]. miR-320a-3p has been found in amniotic fluid and plays a significant role in preventing epithelial–mesenchymal transition (EMT) in lung epithelial cells by targeting connective tissue growth factor (CTGF) and ATG5-associated autophagy [23]. A summary of the most significant effects of the miRNAs we investigated, related to embryo quality, abnormalities, and pregnancy success as reported in earlier studies, is provided in Supplementary Table S2 [3,5,7,8,9,11,16,18,19,20,21,22,23,24,25,26].

## 5. Conclusions

cDNA synthesis can be carried out directly from the medium without prior RNA isolation for qPCR analysis, indicating that miRNAs are actively secreted into the extracellular environment.

Due to the high conservation of miRNA sequences across mammalian species, the human advanced miRNA primers can effectively bind and amplify the corresponding rabbit miRNAs present in the embryo and culture medium.

The observation that most embryo-specific human miRNAs can only be detected in the culture medium of well-developed blastocyst embryos suggests a strong correlation between miRNA secretion and embryonic developmental stage.

Blastocyst-stage embryos have undergone critical cell differentiation and structural organisation, which likely influence the active secretion or release of specific miRNAs into the extracellular environment, involved in key regulatory processes essential for embryo viability, implantation potential, and intercellular communication.

The presence of these miRNAs in the culture medium can serve as an indicator or biomarker of successful embryonic development to the blastocyst stage. As a result of the series of experiments, we determined the miRNAs whose expression levels change in embryos of different stages and quality. Using the literature data, we refined the list of miRNAs that are crucial for the development of an embryo capable of implantation in both rabbit and human samples. Based on our results, we set up a test system that can be used for non-invasive prediction of implantation and pregnancy success in human reproductive medicine.

## Figures and Tables

**Table 1:** Used advanced miRNA primers (Catalogue Number: A25576).

Name of miRNAs	ID Number	miRNA Sequency
mmu-miR-302b-3p	481677_mir	UAAGUGCUUCCAUGUUUUAGUAG
hsa-miR-92a-3p ^#^	477827_mir	UAUUGCACUUGUCCCGGCCUGU
hsa-miR-378a-3p	478349_mir	ACUGGACUUGGAGUCAGAAGGC
hsa-miR-372-3P	478071_mir	AAAGUGCUGCGACAUUUGAGCGU
hsa-miR-371a-5p	478851_mir	ACUCAAACUGUGGGGGCACU
hsa-miR-320a-3p	478594_mir	AAAAGCUGGGUUGAGAGGGCGA
hsa-miR-302c-3P	478509_mir	UAAGUGCUUCCAUGUUUCAGUGG
hsa-miR-28-3p	477999_mir	CACUAGAUUGUGAGCUCCUGGA
hsa-miR-24-3p	47932_mir	UGGCUCAGUUCAGCAGGAACAG
hsa-miR-191-5p	477952_mir	CAACGGAAUCCCAAAAGCAGCUG
hsa-miR-181a-5p	477857_mir	AACAUUCAACGCUGUCGGUGAGU
hsa-miR-103a-3p	478253_mir	AGCAGCAUUGUACAGGGCUAUGA

^#^ endogenous control.

**Table 2:** Preparation of the qPCR TaqMan™ Fast Advanced reaction mix.

Components	Catalogue Numbers	96-Well Plate/Well	Final Concentration/Well
TaqMan™ Fast Advanced Master Mix for qPCR (2×)	Applied Biosystems™/4444556	10 μL	1×
TaqMan Advanced miRNA Assay/miRNA probe (20×)	Applied Biosystems™/A25576	1 μL	1×
cDNA template	Applied Biosystems™/TaqMan™ Advanced miRNA cDNA Synthesis Kit/A28007	5 μL	0.001–100 ng/well
Nuclease-free water	Invitrogen™/AM9938 (Invitrogen, Waltham, CA, USA)	4 μL	
Total		20 μL	

**Table 3:** hsa-miRNA count numbers in different samples, based on SOLiD small RNA library published by Maraghechi et al. in 2013 [10].

Name of miRNAs	MEF (p2)	REF (p2)	rabESC (p2)	Rabbit Embryo (dpc 6)	Rabbit Embryo (dpc 7)	Rabbit PGCs (dpc 14)
mmu-miR-302b-3p	0	0	1	1	4	0
hsa-miR-92a-3p	125	350	95	61	471	1540
hsa-miR-378a-3p	90	48	21	0	13	16
hsa-miR-372-3P	-	-	-	-	-	-
hsa-miR-371a-5p	0	0	0	7	362	0
hsa-miR-320a-3p	70	41	29	6	15	34
hsa-miR-302c-3p	0	0	0	2	7	0
hsa-miR-28-3p	3	8	1	0	0	2
hsa-miR-24-3p	2410	2130	471	3	34	87
hsa-miR-191-5p	462	555	56	7	187	82
hsa-miR-181a-5p	248	96	57	1	3	51
hsa-miR-103a-3p	675	662	55	7	90	77

MEF (p2): 14 days post-coitum (dpc) mouse embryo derived embryonic fibroblast cells at 2nd passage, REF (p2): 14 days post-coitum (dpc) rabbit embryo derived embryonic fibroblast cells at 2nd passage, rabESC (p2): rabbit ES-like cells at 2nd passage, rabbit embryo (dpc 6): 6 days post-coitum (dpc) rabbit embryos (6-day-old), rabbit embryo (dpc 7): 7 days post-coitum (dpc) rabbit embryos (7-day-old), rabbit PGCs (dpc 14): 14dpc genital ridges containing primordial germ cells (PGCs) from 14dpc embryos Quantification was performed based on the comparison of small RNA libraries constructed from total RNA using the SOLiD™ Small RNA Expression Kit.

## Data Availability

Where data supporting reported results can be found, links are included to publicly archived datasets analysed or generated during the study.

## Supplementary Materials

The following supporting information can be downloaded at: https://www.mdpi.com/article/10.3390/genes16091042/s1, Table S1A: List of rabbit embryos cultured in group; Table S1B: List of individually cultured embryos and collected culture medium; Table S2: Proposed functions of the examined miRNAs in relation to embryonic development; Table S3: Summary of used rabbits and examined embryos; Figure S1: 6.5-day-old (6.5 dpc) rabbit embryos developed in vivo in the uterus of a pregnant female and were flushed from the uterus on the 6th day after insemination. We analysed the miRNA expression profile of these embryos using SOLiD sequencing. Movie S1: The rabbit embryo was carefully retrieved from the oviduct of an inseminated female rabbit two days post-insemination. It was then cultured individually in GT-L medium until it reached the blastocyst stage. The development of the embryo was monitored using CytoSMART Lux2 (Lonza Ltd, Germany) time-lapse video microscopy, which captured images every five minutes in a standard culture environment.
