# Supplementary material for: Detection of Development-Specific MicroRNAs in Rabbit Embryos and Culture Media: A Potential Biomarker Approach for Embryo Quality Assessment
Source: Genes (Basel). 2025 Sep 3;16(9):1042. doi: 10.3390/genes16091042 (PMC12469934; doi:10.3390/genes16091042)
Supplement: Supplementary file 1 [file genes-16-01042-s001.zip › genes-3762155-supplementary/genes-3762155_rev_250826/genes-3762155_Rev_SupFig_SupMovies_AppendixA_250810_rev_250826.pdf]

Appendix A:

Detection of Development-Specific MicroRNAs in Rabbit Embryos and Culture Media: A Potential Biomarker Approach for Embryo Quality Assessment

María Salinas[1,2,#], Nikolett Tokodyné Szabadi[1,2,#], Gréta Dévai[1,2], Martin Urbán[1,2], Arnold Tóth[1,2], Bence Lázár[1,2], Timea Pintér [1,2], Annamária Nemes[3], Péter Fancsovits[3], Lilla Bodrogi[1,2], Elen Gócza[1,2*]

[1] Hungarian University of Agriculture and Life Sciences, Institute of Genetics and Biotechnology, Animal Bio-technology Department, Gödöllő, Hungary
[2] Agribiotechnology and Precision Breeding for Food Security National Laboratory, Gödöllő, Hungary
[3] Semmelweis University, Department of Obstetrics and Gynaecology, Division of Assisted Reproduction, Budapest, Hungary
[4] National Centre for Biodiversity and Gene Conservation, Institute for Farm Animal Gene Conservation, Gödöllő, Hungary
* Correspondence: Gocza.Elen@uni-mate.hu
# First authors

Supplementary Table S1A-1B; Supplementary Figure S1-2; Supplementary Movie

Supplementary table S1.A: List of rabbit embryos cultured in group

Sample Identification	Sample type	Number of cultured embryos	Age	Developmental stage
A_II_e; A_II_m	embryo	5	4-day-old	blastocyst - stage 2
F1_m	medium	5	6-day-old	hatched blastocyst - stage 1
F3_m*	medium	5	6-day-old	hatched blastocyst - stage 2

**Reference sample*

Supplementary table S1.B: List of individually cultured embryos and collected culture medium

Sample Identification	Age	Class
A_I_4_e; A_I_4_m	6-day-old	hatched blastocyst - stage 2
A_I_7_e; A_I_7_m	6-day-old	hatched blastocyst - stage 2
A_IV_1_e; A_IV_1_m	6-day-old	hatched blastocyst - stage 3
A_IV_2_e; A_IV_2_m	6-day-old	hatched blastocyst - stage 3
A_IV_3_e; A_IV_3_m	6-day-old	hatched blastocyst - stage 3
A_III_3_e; A_III_3_m	6-day-old	hatched blastocyst - stage 2
B6_m	6-day-old	hatched blastocyst - stage 2
D6_m	6-day-old	blastocyst - stage 2
D5_m	6-day-old	blastocyst - stage 2
D1_m	6-day-old	blastocyst - stage 2
C2_m	6-day-old	blastocyst - stage 2
A7_m	6-day-old	blastocyst - stage 2
E5_m	6-day-old	blastocyst - stage 1
B2_m	6-day-old	blastocyst - stage 1
A3_m	6-day-old	blastocyst - stage 1

Supplementary Table S2: Proposed functions of the examined miRNAs in relation to embryonic development	
miR-103	*miR-103:* Stable in porcine liver and uterus, used for normalization (Mahdipour et al., 2015).
	miR-103a-3p: Regulates embryonic stem cell function, potential therapeutic target (Zhu et al., 2025).
miR-181	*miR-181:* Modulates EMT through TGF-β, Wnt/β-catenin, and NF-κB pathways, linked to cancer cell behaviour (Yang et al., 2025).
	miR-181a-5p: Higher in non-developing embryos; associated with oestrogen signalling and implantation; regulates MMP14 affecting cell adhesion and proliferation (Kamijo et al., 2022; Lázár et al., 2018).
miR-191	*miR-191-5p:* Elevated in aneuploid embryos and failed IVF; linked to cell adhesion and successful implantation (Hawke et al., 2021; Kamijo et al., 2022).
	miR-191: Expression varies with embryo viability and pathology (e.g., preeclampsia, diabetes); insulin-regulated; lineage-specific effects in embryoblast vs. trophoblast cells (Mutia et al., 2023; Pendzialek et al., 2019).
miR-24	*miR-24-3p:* Regulates ESC differentiation; inhibits apoptosis via target gene P27; supports follicular health (Mutia et al., 2023; Shi et al., 2024).
	miR-24: Highly expressed in euploid embryos and polycystic ovaries (Rosenbluth et al., 2013; Zhou & Dimitriadis, 2020).
miR-28	*miR-28-3p:* High in good-quality rabbit blastocysts; supports implantation; influences cancer-related genes (Kamijo et al., 2022; Kotarski et al., 2025).
miR-320	*miR-320:* Highly expressed in early euploid embryos; target: ITGB5; linked to vascular development defects when overexpressed (Caporali & Emanueli, 2011; Rosenbluth et al., 2013).
	miR-320a: Higher in non-pregnancy groups; may negatively affect implantation; associated with CDH and lung fibrosis (Kamijo et al., 2022; Timofeeva et al., 2025).
miR-378	*miR-378a-5p:* Influences trophoblast cell invasion and migration in placentation (Mutia et al., 2023).
	miR-378-3p: Linked to oocyte meiosis, blastocyst quality, embryo hatching, and implantation success (Kamijo et al., 2022; Pavani et al., 2022).

	miR-378: Highly expressed in euploid embryos; associated with sperm motility and quality (Rosenbluth et al., 2013; Turri et al., 2021).
miR-92	*miR-92:* Found in polycystic ovaries (Zhou & Dimitriadis, 2020).
	miR-92a-3p: Regulates genes involved in cell adhesion, important for implantation; may serve as a biomarker for embryo viability (Kamijo et al., 2022).
	miR-92a: Highly expressed in euploid embryos; overexpression causes vascular defects (Caporali & Emanueli, 2011; Rosenbluth et al., 2013).

References:

- Caporali, A., & Emanueli, C. (2011). MicroRNA regulation in angiogenesis. In *Vascular Pharmacology* (Vol. 55, Issue 4, pp. 79–86). https://doi.org/10.1016/j.vph.2011.06.006
- Hawke, D. C., Watson, A. J., & Betts, D. H. (2021). Extracellular vesicles, microRNA and the preimplantation embryo: non-invasive clues of embryo well-being. *Reproductive BioMedicine Online*, *42*, 39–54. https://doi.org/10.1016/j
- Kamijo, S., Hamatani, T., Sasaki, H., Suzuki, H., Abe, A., Inoue, O., Iwai, M., Ogawa, S., Odawara, K., Tanaka, K., Mikashima, M., Suzuki, M., Miyado, K., Matoba, R., Odawara, Y., & Tanaka, M. (2022). MicroRNAs secreted by human preimplantation embryos and IVF outcome. *Reproductive Biology and Endocrinology*, *20*(1). https://doi.org/10.1186/s12958-022-00989-0
- Kotarski, K., Kot, M., & Skrzypek, K. (2025). miR-28: A Tiny Player in Cancer Progression and Other Human Diseases. *Biomolecules*, *15*(6), 757. https://doi.org/10.3390/biom15060757
- Lázár, B., Anand, M., Tóth, R., Várkonyi, E. P., Liptói, K., & Gócza, E. (2018). Comparison of the MicroRNA Expression Profiles of Male and Female Avian Primordial Germ Cell Lines. *Stem Cells International*, *2018*, 1–17. https://doi.org/10.1155/2018/1780679
- Mahdipour, M., Van Tol, H. T. A., Stout, T. A. E., & Roelen, B. A. J. (2015). Validating reference microRNAs for normalizing qRT-PCR data in bovine oocytes and preimplantation embryos. *BMC Developmental Biology*, *15*(1). https://doi.org/10.1186/s12861-015-0075-8
- Mutia, K., Wiweko, B., Abinawanto, A., Dwiranti, A., & Bowolaksono, A. (2023). microRNAs as A Biomarker to Predict Embryo Quality Assessment in In Vitro Fertilization. In *International Journal of Fertility and Sterility* (Vol. 17, Issue 2, pp. 85–91). Royan Institute (ACECR). https://doi.org/10.22074/ijfs.2022.551571.1285

- Pavani, K. C., Meese, T., Pascottini, O. B., Guan, X., Lin, X., Peelman, L., Hamacher, J., Nieuwerburgh, F. Van, Deforce, D., Boel, A., Orn Heindryckx, B., Tilleman, K., Soom, A. Van, Gadella, B. M., Hendrix, A., & Smits, K. (2022). *Hatching is modulated by microRNA-378a-3p derived from extracellular vesicles secreted by blastocysts*. https://doi.org/10.1073/pnas
- Pendzialek, S. M., Knelangen, J. M., Schindler, M., Gürke, J., Grybel, K. J., Gocza, E., Fischer, B., & Navarrete Santos, A. (2019). Trophoblastic microRNAs are downregulated in a diabetic pregnancy through an inhibition of Drosha. *Molecular and Cellular Endocrinology*, *480*, 167–179. https://doi.org/10.1016/j.mce.2018.11.002
- Rosenbluth, E. M., Shelton, D. N., Sparks, A. E. T., Devor, E., Christenson, L., & Van Voorhis, B. J. (2013). MicroRNA expression in the human blastocyst. *Fertility and Sterility*, *99*(3). https://doi.org/10.1016/j.fertnstert.2012.11.001
- Shi, S., Zhang, L., Wang, L., Yuan, H., Sun, H., Madaniyati, M., Cai, C., Pang, W., Gao, L., & Chu, G. (2024). miR-24-3p promotes proliferation and inhibits apoptosis of porcine granulosa cells by targeting P27. *Journal of Integrative Agriculture*, *23*(4), 1315–1328. https://doi.org/10.1016/j.jia.2023.04.008
- Timofeeva, A. V., Fedorov, I. S., Naberezhnev, Y. I., Tetruashvili, N. K., & Sukhikh, G. T. (2025). Key Amniotic Fluid miRNAs as Promising Target Molecules for the Antenatal Prevention of Pulmonary Hypoplasia Associated with Congenital Diaphragmatic Hernia. *International Journal of Molecular Sciences*, *26*(8). https://doi.org/10.3390/ijms26083872
- Turri, F., Capra, E., Lazzari, B., Cremonesi, P., Stella, A., & Pizzi, F. (2021). A Combined Flow Cytometric Semen Analysis and miRNA Profiling as a Tool to Discriminate Between High- and Low-Fertility Bulls. *Frontiers in Veterinary Science*, *8*. https://doi.org/10.3389/fvets.2021.703101
- Yang, C., Wang, R., & Hardy, P. (2025). The Multifaceted Roles of MicroRNA-181 in Stem Cell Differentiation and Cancer Stem Cell Plasticity. In *Cells* (Vol. 14, Issue 2). Multidisciplinary Digital Publishing Institute (MDPI). https://doi.org/10.3390/cells14020132
- Zhou, W., & Dimitriadis, E. (2020). Secreted MicroRNA to Predict Embryo Implantation Outcome: From Research to Clinical Diagnostic Application. In *Frontiers in Cell and Developmental Biology* (Vol. 8). Frontiers Media S.A. https://doi.org/10.3389/fcell.2020.586510
- Zhu, M., Chu, Y., Yuan, Q., Li, J., Chen, S., & Li, L. (2025). Integrated bioinformatics analysis to explore potential therapeutic targets and drugs for small cell carcinoma of the esophagus. *Frontiers in Bioinformatics*, *5*. https://doi.org/10.3389/fbinf.2025.1495052

Supplementary Table S3. Summary of used rabbits and examined embryos

	Experiment 1	*Experiment 2*	Total
Number of used sperm donor males	*4*		**4**
Number of used embryo donor females	4	4	**8**
Number of collected embryos	30	31	**61**
Number of cultured embryos	28	24	**52**
Number of examined blastocysts	28	20	**48**

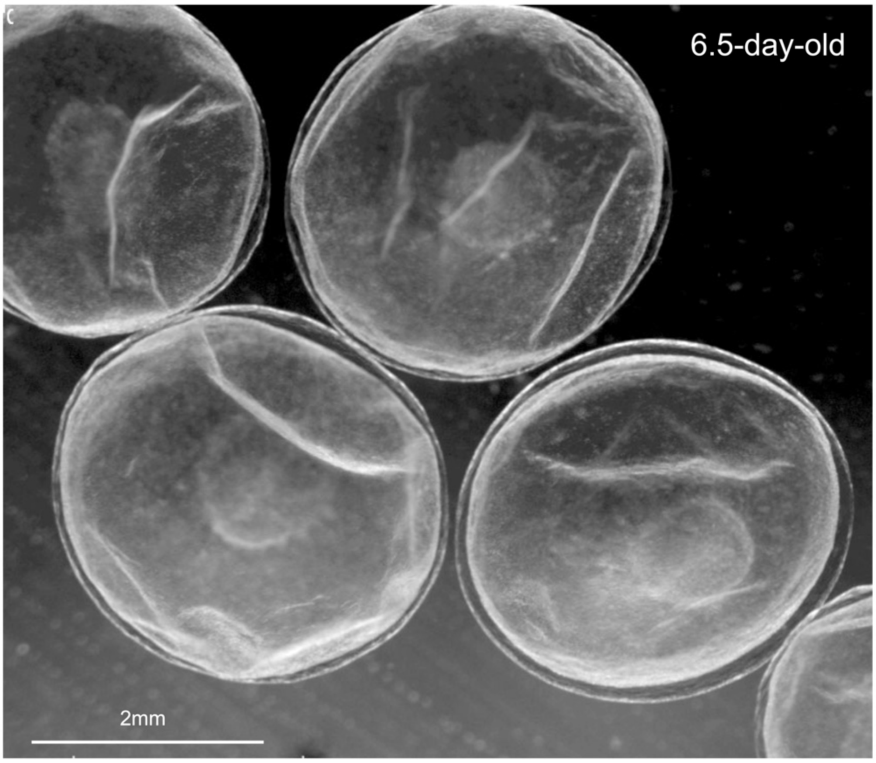

Supplementary Figure S1:

6.5-day-old (6.5 dpc) rabbit embryos developed *in vivo* in the uterus of a pregnant female and were flushed from the uterus on the 6th day after insemination. We analysed the miRNA expression profile of these embryos using SOLiD sequencing.

Supplementary Movie S1:

The rabbit embryo was carefully retrieved from the oviduct of an inseminated female rabbit two days post-insemination. It was then cultured individually in GT-L medium until it reached the blastocyst stage. The development of the embryo was monitored using CytoSMART Lux2 (Lonza Ltd, Germany) time-lapse video microscopy, which captured images every five minutes in a standard culture environment.
