# Supplementary figures and images for: Detection of Development-Specific MicroRNAs in Rabbit Embryos and Culture Media: A Potential Biomarker Approach for Embryo Quality Assessment
Source: Genes (Basel). 2025 Sep 3;16(9):1042. doi: 10.3390/genes16091042 (PMC12469934; doi:10.3390/genes16091042)

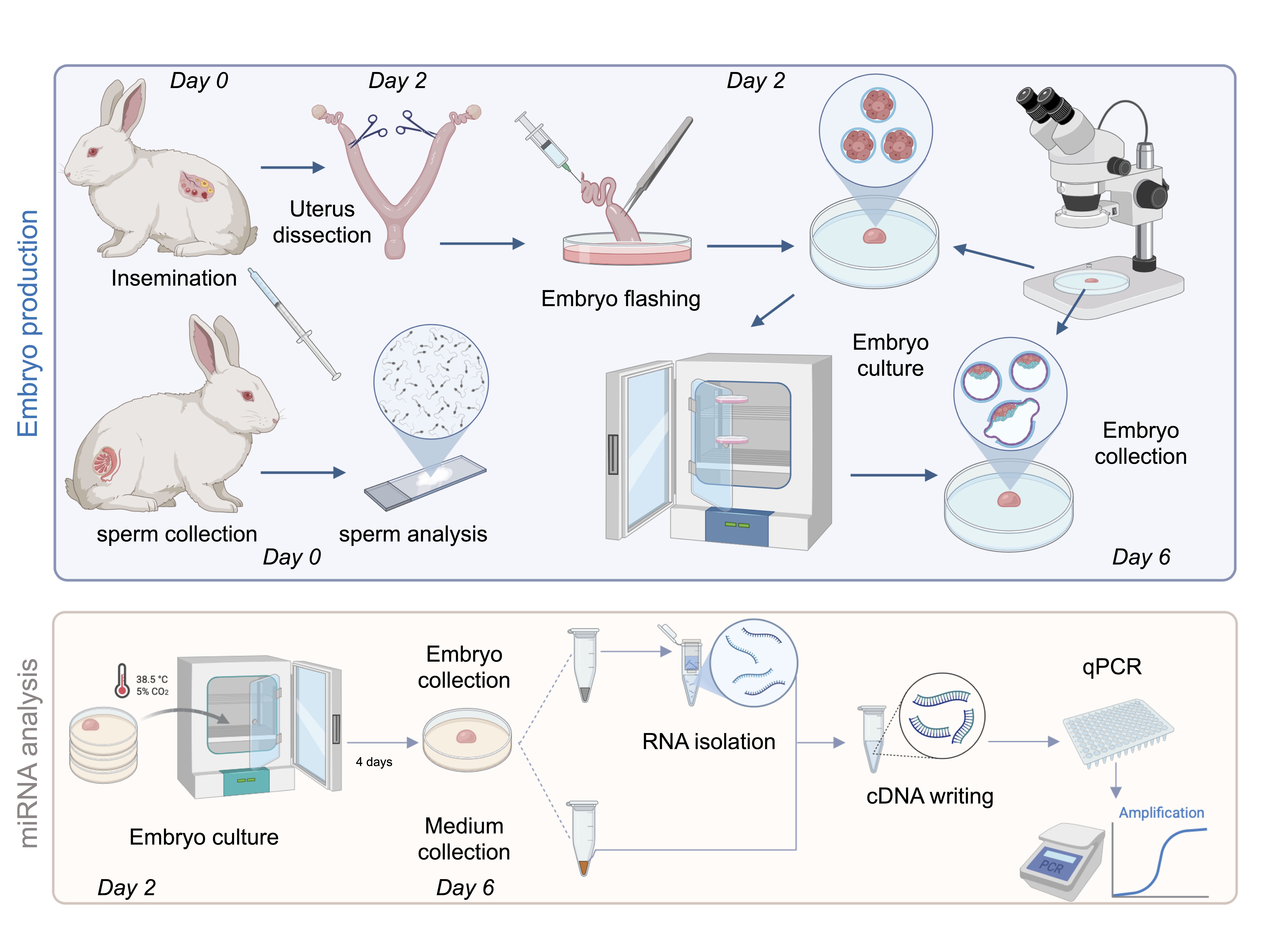

Supplement: Supplementary file 1 [file genes-16-01042-s001.zip › genes-3762155-supplementary/genes-3762155_rev_250826/genes-3762155_Figure1_final.jpg]

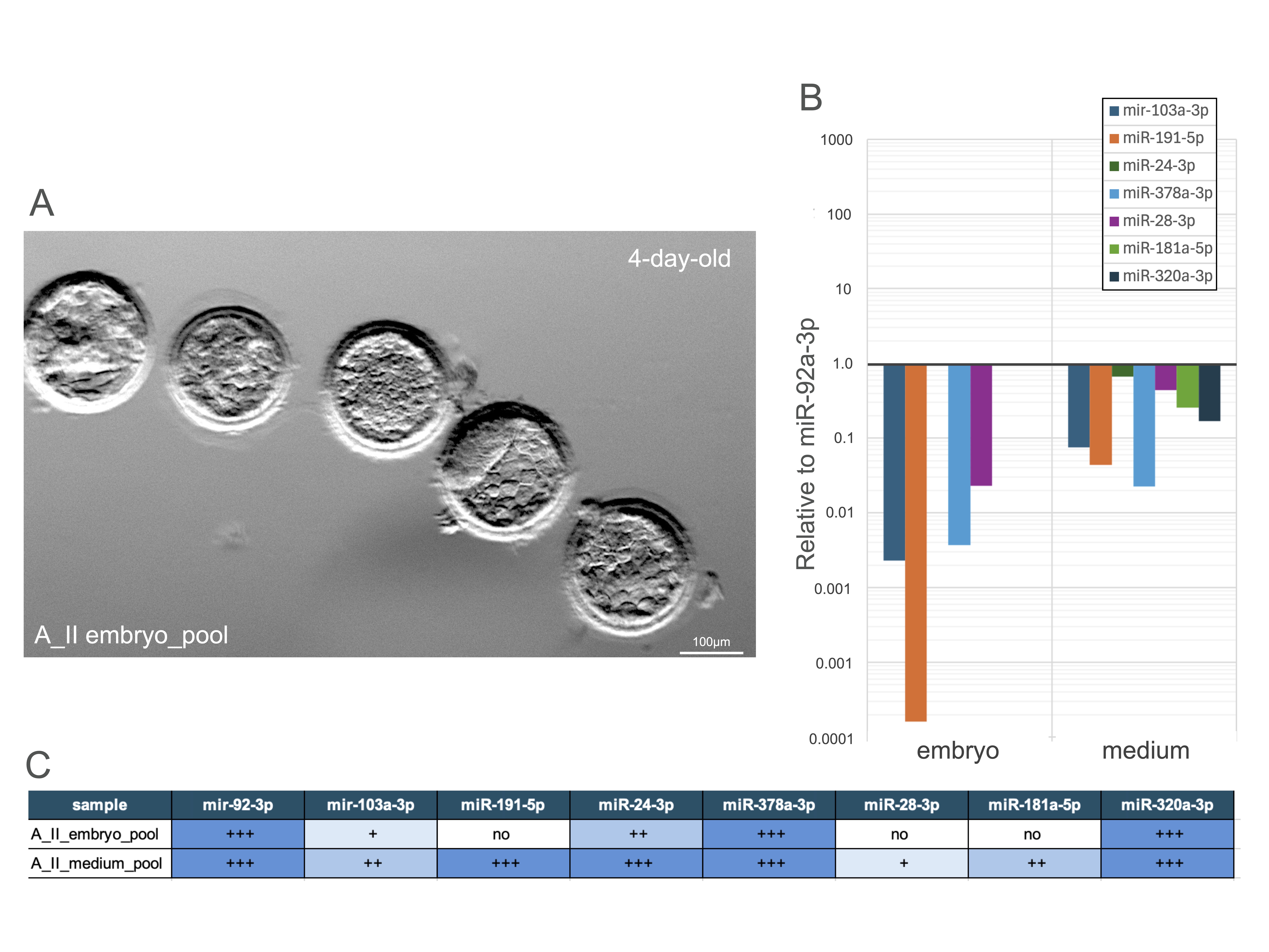

Supplement: Supplementary file 1 [file genes-16-01042-s001.zip › genes-3762155-supplementary/genes-3762155_rev_250826/genes-3762155_Figure2_1_final.jpg]

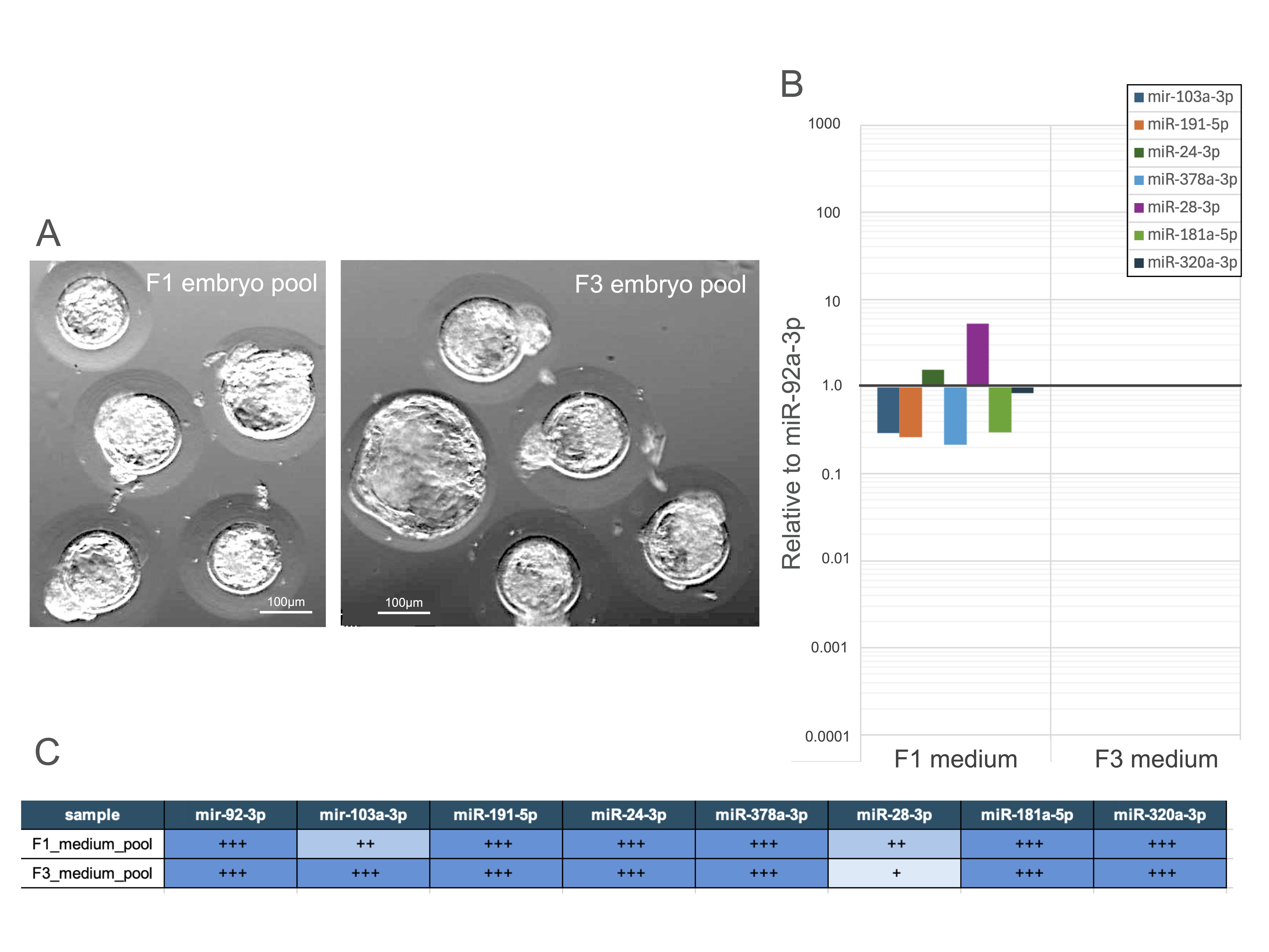

Supplement: Supplementary file 1 [file genes-16-01042-s001.zip › genes-3762155-supplementary/genes-3762155_rev_250826/genes-3762155_Figure2_2_final.jpg]

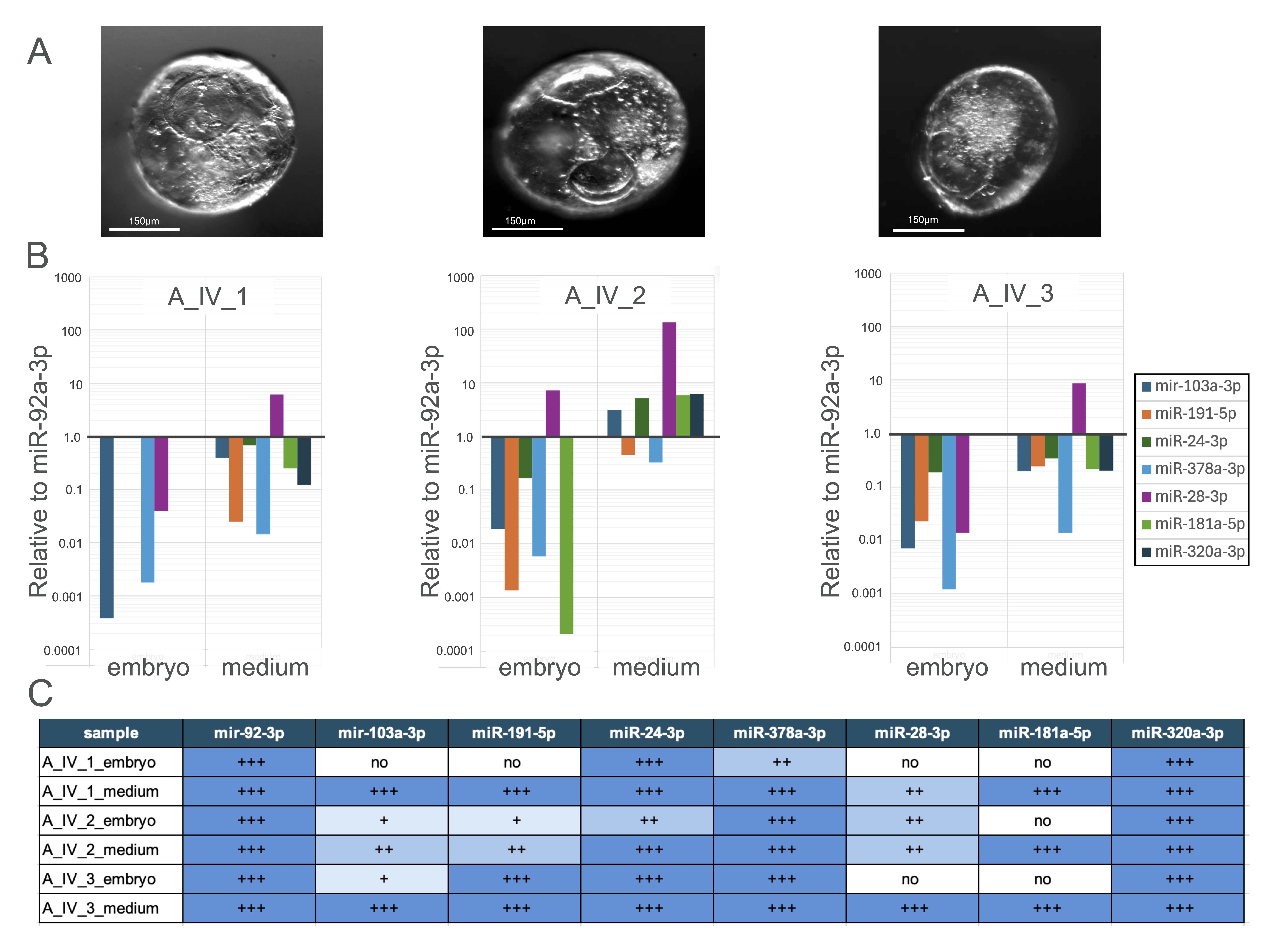

Supplement: Supplementary file 1 [file genes-16-01042-s001.zip › genes-3762155-supplementary/genes-3762155_rev_250826/genes-3762155_Figure3_final.jpg]

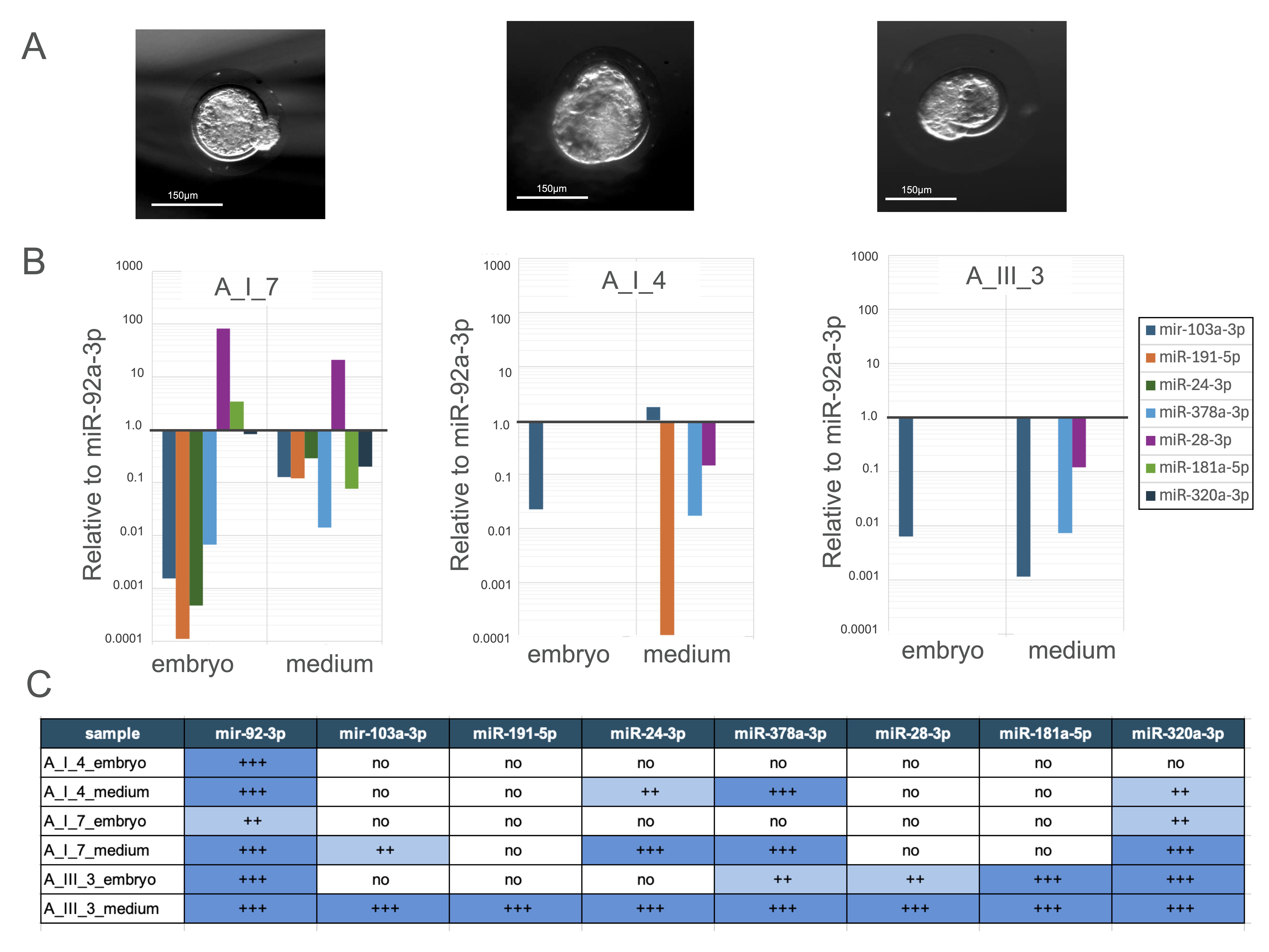

Supplement: Supplementary file 1 [file genes-16-01042-s001.zip › genes-3762155-supplementary/genes-3762155_rev_250826/genes-3762155_Figure4_final.jpg]

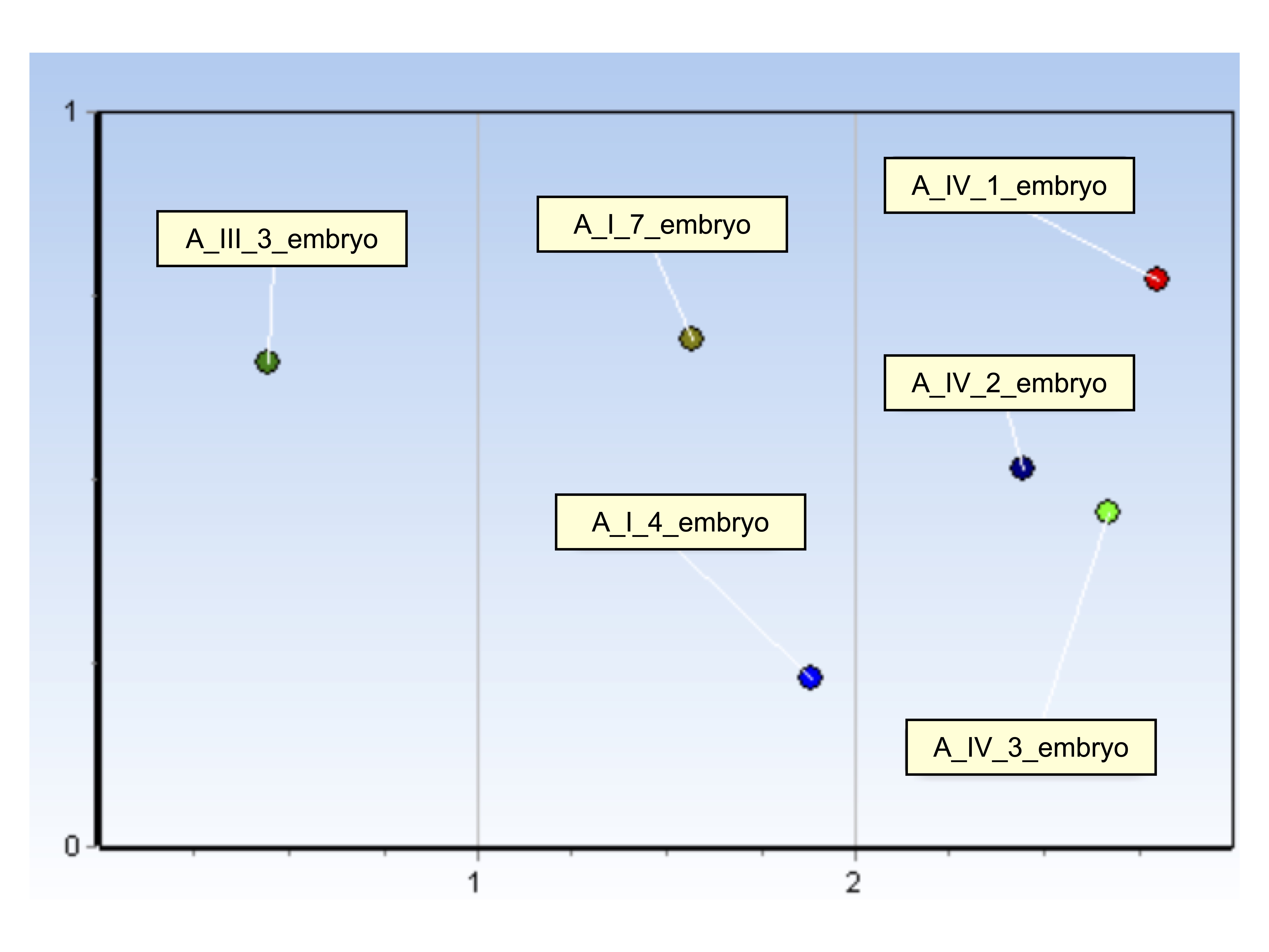

Supplement: Supplementary file 1 [file genes-16-01042-s001.zip › genes-3762155-supplementary/genes-3762155_rev_250826/genes-3762155_Figure5_final.jpg]

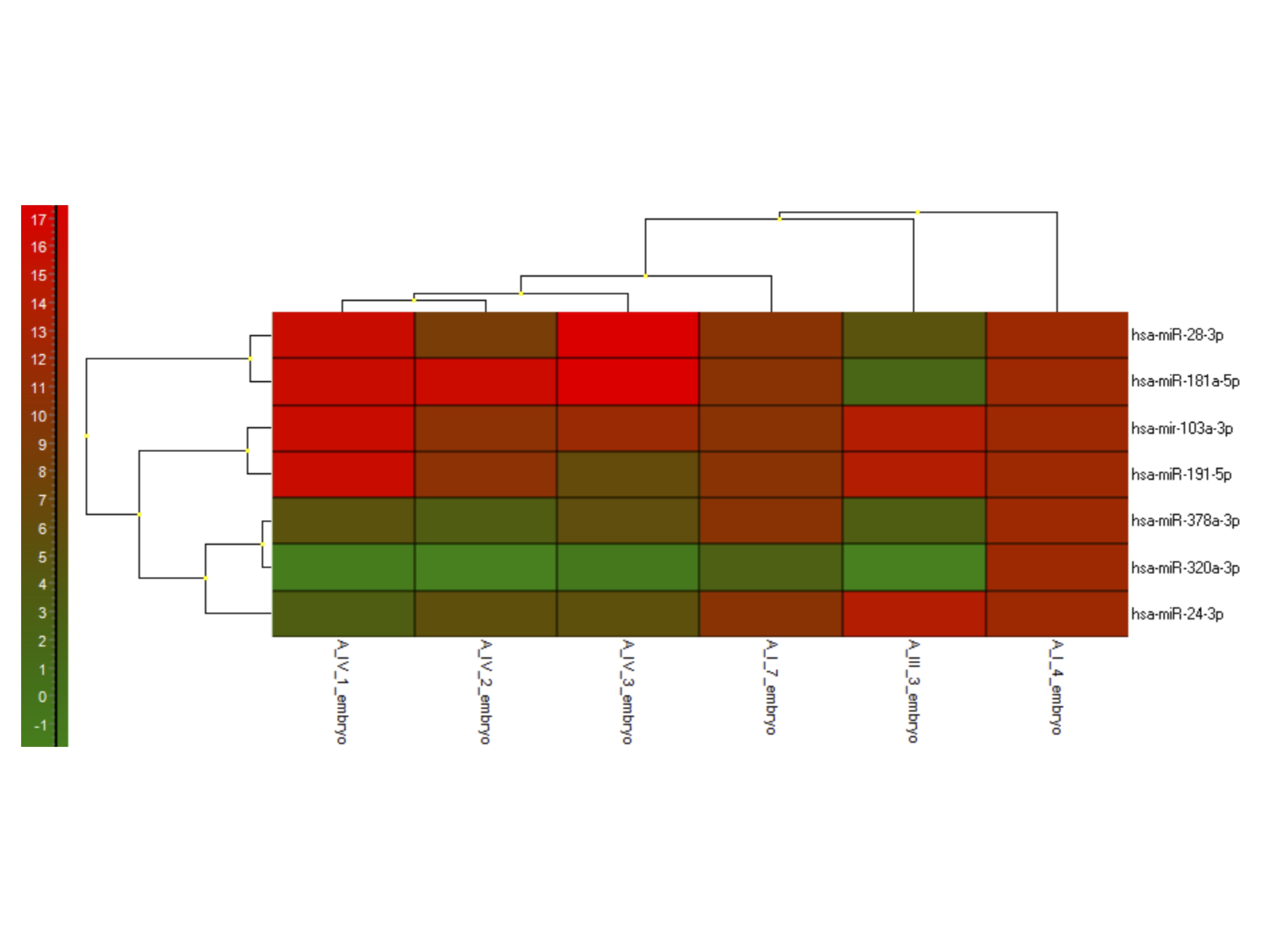

Supplement: Supplementary file 1 [file genes-16-01042-s001.zip › genes-3762155-supplementary/genes-3762155_rev_250826/genes-3762155_Figure6_final.jpg]

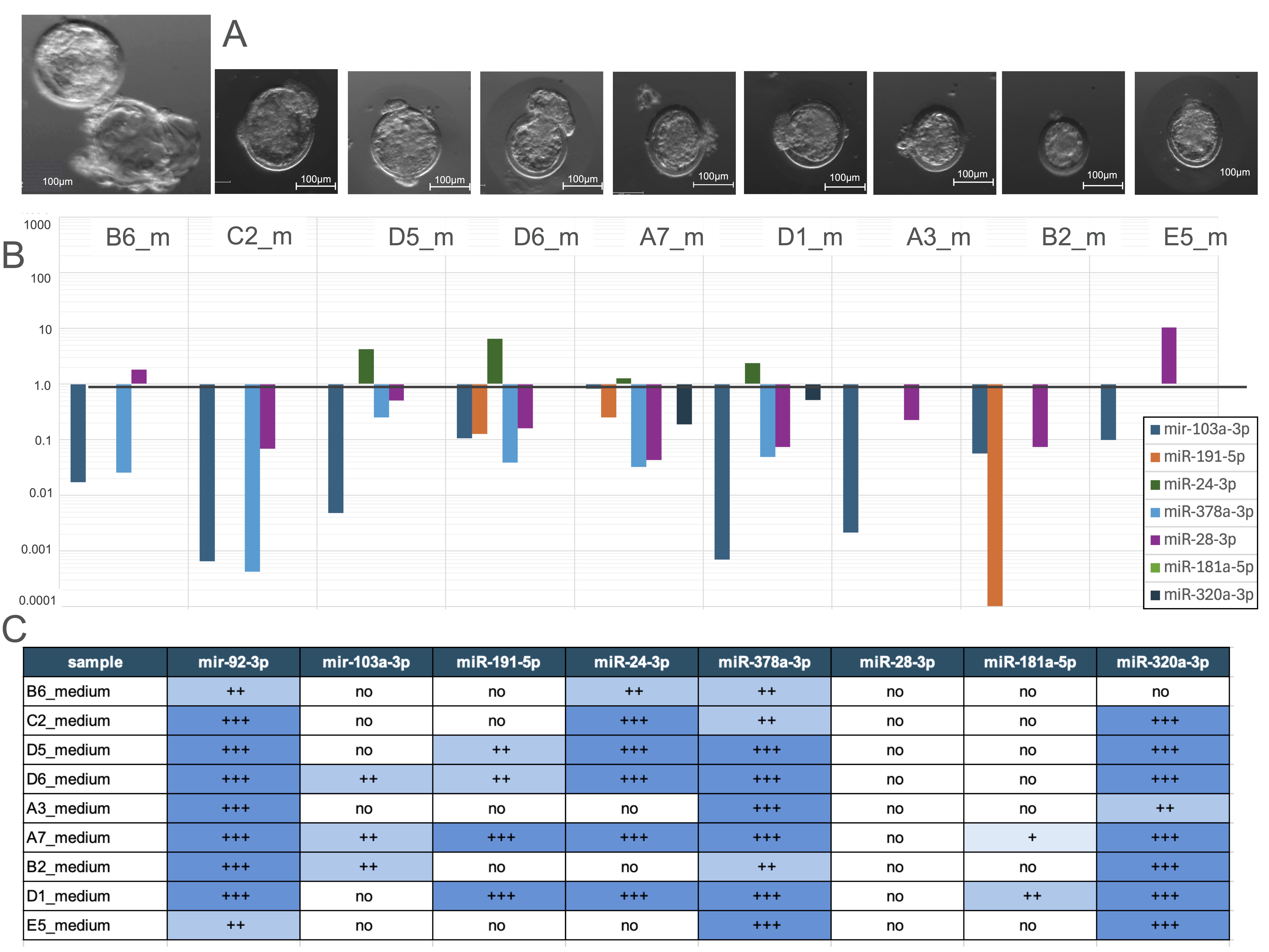

Supplement: Supplementary file 1 [file genes-16-01042-s001.zip › genes-3762155-supplementary/genes-3762155_rev_250826/genes-3762155_Figure7_final.jpg]

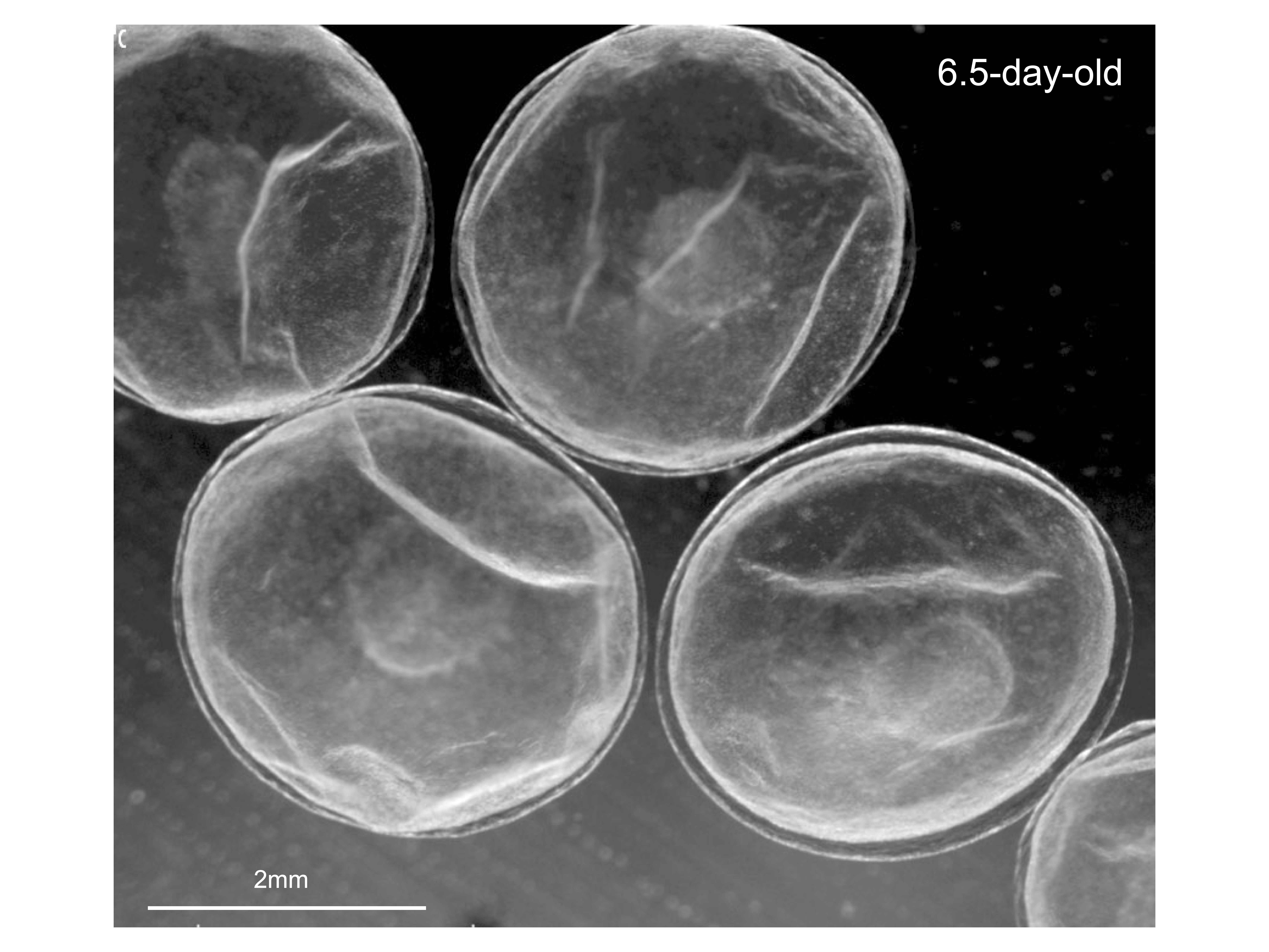

Supplement: Supplementary file 1 [file genes-16-01042-s001.zip › genes-3762155-supplementary/genes-3762155_rev_250826/genes-3762155_SupFig1_250810.jpg]
